# A Systematic Review of the Patterns of Associative Multimorbidity in Asia

**DOI:** 10.1155/2021/6621785

**Published:** 2021-07-03

**Authors:** Shawn S. Rajoo, Zhi Jie Wee, Poay Sian Sabrina Lee, Fang Yan Wong, Eng Sing Lee

**Affiliations:** ^1^Yong Loo Lin School of Medicine, National University of Singapore, Singapore; ^2^Clinical Research Unit, National Healthcare Group Polyclinics, Singapore; ^3^Lee Kong Chian School of Medicine, Nanyang Technological University, Singapore

## Abstract

Patients with multimorbidity are commonly seen in primary care. An increasing number of multimorbidity patterns are being reported in the Western literature with a few from Asia. The main objective of this systematic review was to describe patterns of associative multimorbidity, defined as associations beyond chance or patterns of diseases, in the Asian population. We searched Medical Literature Analysis and Retrieval System Online (MEDLINE (Ovid)), Excerpta Medica Database (EMBASE), Cumulative Index to Nursing and Allied Health Literature (CINAHL), Web of Science (Clarivate Analytics), and Scopus (Elsevier) databases from their inception to April 22, 2019 using medical subject headings, keywords in titles, abstracts, and text. We used the Modified Newcastle-Ottawa Scale for risk-of-bias assessment. Eight articles from China, India, Indonesia, and Japan met the inclusion criteria. Patterns of associative multimorbidity were reported as dyadic/triadic disease combinations or disease clusters. The most common multimorbidity pattern, “cardiovascular and metabolic diseases,” was identified in six of eight articles. The other four multimorbidity patterns are comprised of “mental health problems,” “degenerative diseases,” pulmonary diseases,” and “cancer diseases.” The eight articles showed methodological heterogeneity in terms of the list of chronic diseases, ascertainment of multimorbidity, statistical methods, and study populations. This systematic review identified five common patterns of associative multimorbidity in Asia. “Cardiovascular and metabolic diseases” and “mental diseases” were two patterns that were similarly reported in the Western world. Alignment of the definition of multimorbidity and the statistical methodology are needed to identify the unique patterns of multimorbidity in Asia so that clinical practice guidelines on multimorbidity can be developed for the Asian population.

## 1. Introduction

Multimorbidity is defined as the simultaneous occurrence of multiple chronic diseases in an individual [1, 2]. Individuals with multimorbidity have substantial treatment burdens [3], contributed by polypharmacy, multiple appointments, and treatment regimens for their multiple chronic diseases [4, 5].

This poses a significant challenge on the primary healthcare system since individuals with multimorbidity are mostly managed by primary care physicians [6]. Current clinical guidelines are single-disease focused and are unable to address the needs of individuals with multimorbidity due to the complex interactions of multiple chronic diseases [7].

The National Institute for Health and Care Excellence (NICE) [8] recommends to go beyond the benefits and risks of the single-disease guidelines, while focusing on the interaction between health conditions, treatment, and their effect on the quality of life for multimorbidity. However, it will be impractical to create guidelines for all the permutations and combinations of the multiple chronic diseases.

Thus, there is a need to understand the common patterns of multimorbidity defined as chronic diseases that cluster together most frequently [9]. There were studies which describe disease clusters that occur with the highest frequency or prevalence [10]. However, it may be more meaningful to focus on the associations beyond chance or patterns of diseases, known as associative multimorbidity [11]. Associative multimorbidity is derived by different statistical methodologies, such as observed to expected ratios or odds ratios among the most commonly dyads or triads of chronic conditions, or cluster and factor analyses to identify systematic clusters among diseases.

A systematic review by Prados-Torres et al. identified 14 studies conducted in Western countries on associative multimorbidity [11]. The authors found 63 multimorbidity patterns that were composed of three or more diseases. The three most common patterns were a combination of cardiovascular and metabolic diseases, a combination related with mental health problems, and a combination with musculoskeletal diseases. However, patterns of multimorbidity in the Western population may be different from Asia due to cultural, genetic, and other unknown reasons.

Hence, we conducted a systematic review to describe the patterns of associative multimorbidity in Asia. The specific objectives of this review were to firstly describe the main characteristics and statistical methodologies of the selected studies and secondly identify similarities and differences regarding the patterns of associative multimorbidity identified in the different studies.

## 2. Materials and Methods

We conducted a systematic review to identify the patterns of associative multimorbidity in individuals from the primary healthcare or the general population in Asian countries. We followed the PRISMA statement [12] for reporting this systematic review.

### 2.1. Search Strategy

A study protocol was developed using PRISMA guidelines and published in PROSPERO (PROSPERO registration number: CRD42019129327, http://www.crd.york.ac.uk/PROSPERO/display_record.php?ID=CRD42019129327). Medical Literature Analysis and Retrieval System Online (MEDLINE (Ovid)), Excerpta Medica Database (EMBASE), Cumulative Index to Nursing and Allied Health Literature (CINAHL), Web of Science (Clarivate Analytics), and Scopus (Elsevier) were searched to identify relevant articles, based on Medical Subject Headings (MeSH) and keywords in titles, abstracts, and full texts, from their inception to April 22, 2019. The search strategy used MeSH terms and keywords were “patterns,” multimorbidity,” primary care,” general population,” countries in Asia,” and “comorbidity.” “Comorbidity” was used as a MeSH term because “multimorbidity” was only introduced in 2018 [13] and “comorbidity” was used synonymously with “multimorbidity” before 2018 [14]. The search was restricted to articles published in English only. SSR and ZJW conducted the searches after developing the search strategy in consultation with the librarian at the National University Singapore and reviewed by the other authors. In addition, a “snowball” search on the references of the selected articles was performed by SSR and ZJW. The final search strategy is presented in Appendix S1.

### 2.2. Study Selection

We included articles which reported the patterns of associative multimorbidity in primary care or the general population in Asia. Exclusion criteria were (a) nonoriginal research articles (reviews, editorials, commentaries, protocols, or conference proceedings), (b) articles which described frequency of patterns of multimorbidity without any statistical analysis, (c) articles which recruited participants based on a specific chronic condition or presence of an indexed condition, and (d) articles which defined multimorbidity with less than 12 chronic conditions [15]. Although the definition and criteria of multimorbidity are still contentious, we adopted the suggestion by Fortin et al. to use a list of at least 12 chronic conditions to form the multimorbidity list of chronic conditions [15].

### 2.3. Eligibility Assessment and Data Extraction

Two study team members (SSR and ZJW) assessed the titles, abstracts, and full texts of the original articles for eligibility. Any disagreements in this process were resolved by consensus or when necessary, through a third study team member (ESL). Relevant information from the selected articles was extracted by SSR and ZJW using a data extraction form that was piloted and improved on. SSR and ZJW also independently assessed the risk of bias of the selected articles using the Modified Newcastle-Ottawa Scale (Appendix S2) [16] assessing (i) representativeness of the sample, (ii) ascertainment of multimorbidity, and (iii) appropriateness of the statistical analysis. The modifications on the Newcastle-Ottawa Scale [16] included addition of “sample size” and “non-respondents” under “representativeness of the sample population,” change “ascertainment of exposure” to “ascertainment of multimorbidity,” and “assessment of outcome” to “appropriateness of the statistical analysis.” We removed two components—"comparability” and “was follow-up long enough for outcomes to occur”—because they were not relevant for assessing quality for this systematic review. For information which could not be obtained from the final eight articles selected [17–24], SSR and ZZJ emailed the authors for further clarification. In particular, details regarding the statistical analysis in two articles [19, 23] were clarified with the authors prior to making an objective assessment of the article's risk of bias. Any disagreements on the risk of bias assessments of the articles were resolved by consensus and, if necessary, mediated by the other study team members (PSSL, FYW, and ESL).

## 3. Results and Discussion

**Figure 1:**
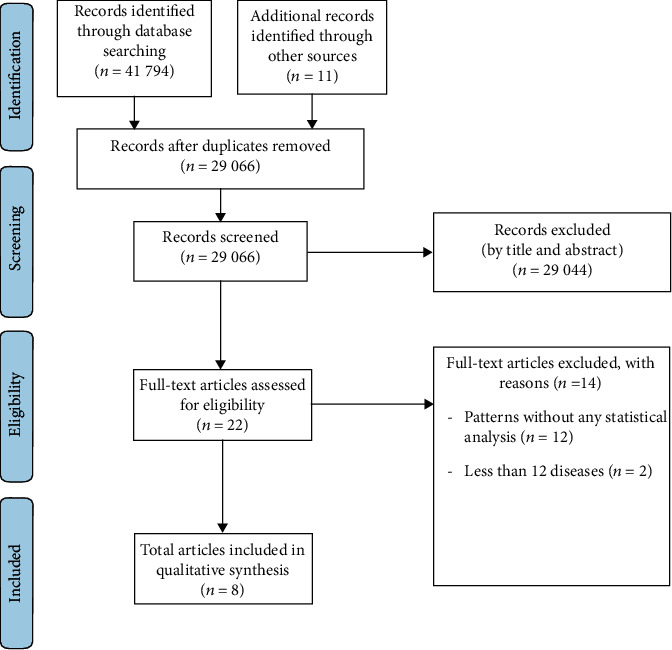
PRISMA flow diagram.

Both electronic and manual searches yielded 41805 articles, of which 12739 were duplicates and 29044 were irrelevant, on the basis of their title and abstract. This resulted in the remaining 22 articles that were available for full-text screening. Ultimately, eight articles were selected [17–24], corresponding to seven studies (Figure 1). Two articles included results from a similar study [21, 22] and another article reported results from two different samples [17].

The sample size of the selected articles ranged from 411 [22] to 21435 [20] participants. Of the eight articles, five of them [19–22, 24] were conducted in China, one from Indonesia [18], one from Japan [23], and one included both from China and India [17] (Table 1).

All eight articles reported cross-sectional designs with various types of sampling strategies. Majority of the articles obtained data from the general population [17–20, 23, 24] except two articles which obtained data from patients attending primary care settings [21, 22]. Three articles [17, 20, 23] used 18 years old and above as the age inclusion criterion while the remaining studies targeted older patients: 40 years old and above [18], 50 years old and above [24], and 60 years old and above [19, 21, 22] (Table 1).

All eight articles defined multimorbidity as the simultaneous occurrence of two or more chronic diseases in an individual. Wang et al. included the duration of the chronic conditions, i.e., “past 12 months,” in their definition of multimorbidity [20]. Data collection methods for measuring multimorbidity were varied. There were self-reported questionnaires [18, 21–24], structured interviews [17, 19–22, 24], clinical examinations or physician's assessments [17–22], and laboratory tests [19]. No studies used administrative data sources as their data collection method. The number of chronic diseases for defining multimorbidity ranged from 12 [17] to 18 [20] (Table 1).

For statistical analysis used to determine the patterns of associative multimorbidity, three articles [17, 22, 23] used exploratory factor analysis, two articles [19, 21] used both the observed/expected ratio and exploratory factor analysis, and each of the three remaining articles used the observed/expected ratio [18], logistic regression [20], and hierarchical cluster analysis [24] individually. Articles which used exploratory factor analysis or hierarchical cluster analysis reported disease clusters [17, 19, 21–24] for the patterns of multimorbidity, whereas dyadic/triadic disease combinations [18–21] were reported in articles which used the observed/expected ratio or logistic regression (Table 2).

Five of the articles were found to have overall low risk of bias using the Modified Newcastle-Ottawa Scale. A major reason for the two articles with high risk of bias [23, 24] and one article with medium risk of bias [18] were on the component of “ascertainment of multimorbidity” where the authors relied on responses from self-reported questionnaires only to determine individuals with multimorbidity without corroboration with a physician's assessment or clinical examination (Table 3).

The following similarities were found in the patterns of associative multimorbidity from the eight articles. The first associative multimorbidity pattern, “cardiovascular and metabolic diseases,” was identified in six of eight articles [17, 19, 21–24]. This pattern was described as *cardiorespiratory*, *metabolic*, *cardiopulmonary-mental-degenerative disorders*, *cerebrovascular-metabolic disorders*, *cardiovascular and metabolic disorders*, *cardiovascular/renal/metabolic*, *vascular-metabolic*, and/or *hepatorenal*. The most common diseases found in this pattern were hypertension (6 articles), diabetes (6 articles), dyslipidemia (5 articles), coronary heart disease (4 articles), kidney disease (4 articles), stroke (3 articles), and obesity (2 articles). Additional diseases related to respiratory, mental, and degenerative disorders were also identified (Table 4).

The second associative multimorbidity pattern identified in four of eight articles was “mental health problems” [17, 19, 23, 24]. The most common disease found in this pattern was depression, which was found in two of the four articles [17, 19]. The other two articles [23, 24] used a generic term of “*mental disorders or emotional*, *nervous or psychiatric problems*” to describe the diseases present in their patterns (Table 4).

The third associative multimorbidity pattern, “degenerative diseases,” was identified in six of eight articles [17, 19, 21–24], described as *mental-articular*, *cardiopulmonary-mental-degenerative disorders*, *degenerative disorders*, and *skeletal/articular/digestive*, *cognitive-emotional.* The most common diseases found in this pattern were arthritis or joint disease (4 articles), cataract or eye problems (3 articles), and hearing disorders (3 articles). The diseases identified are associated with advance age and involve a wide variety of chronic diseases such as cancer, cardiometabolic disorders, and mental disorders (Table 4).

“Pulmonary diseases” were the fourth associative multimorbidity pattern identified in five articles [17, 19, 21–23]. The authors described the patterns as follows: *cardiorespiratory*, *cardiopulmonary-mental-degenerative disorders*, *digestive and respiratory disorders*, or *respiratory/dermal*. The most common respiratory diseases found in this pattern were asthma and chronic pulmonary obstructive disorder (2 articles). The other three articles used a generic term of “*lung diseases*” (2 articles) and “*chronic respiratory diseases*” (1 article). Additional diseases related to gastrointestinal (2 articles) and cardiometabolic disorders (2 articles) were also identified (Table 4).

Lastly, “cancer diseases” were the fifth associative multimorbidity pattern identified in three articles [21–23], described as “*cancer*” (2 articles) and “*malignancy*” (1 article). Additional diseases related to degenerative diseases such as cataract, joint disease, and hearing disorders were also identified (Table 4).

## 4. Discussion

This systematic review included eight articles from seven studies in four different Asian countries (China, India, Japan, and Indonesia). Patterns of associative multimorbidity were reported as dyadic/triadic disease combinations and disease clusters. Out of the eight articles, four different statistical methods were used to describe associative multimorbidity patterns (exploratory factor analysis, observed/expected ratio, logistic regression, and hierarchical cluster analysis). Of the four methods, exploratory factor analysis was the most common and was used in five of the eight articles [17, 19, 21–23]. The most common disease patterns included “cardiovascular and metabolic diseases” as well as “degenerative diseases.”

### 4.1. Nonuniform Methodology in Articles Selected

It was notable that despite the small number of Asian studies included for the systematic review, there were several methodologies used. The differences in methodologies made comparison between the studies difficult [15].

Firstly, each of the eight articles included in this systematic review varied in terms of the actual list of chronic diseases as well as the way the chronic diseases were selected. The number of chronic diseases considered for defining multimorbidity ranged from 12 to 18. Only two out of the eight articles [17, 20] reported the selection criteria for the chronic diseases. The other articles did not specify how the list of chronic diseases was selected.

Secondly, the ascertainment of multimorbidity also contributed to the variation between studies. Of the eight articles, we noted that the data sources from which chronic diseases were identified differed from study to study. For example, while six of the eight articles [17–22] relied on a combination of self-reported physician's diagnoses and active measurements (such as blood pressure, height, and weight), two articles [23, 24] depended entirely on written self-reported data. This could have resulted in under- or overidentification of chronic diseases amongst the respondents [25]. Hussain et al. used a unique method in the ascertainment of multimorbidity [18]. In the study, respondents with no reported diabetes or heart problems, undiagnosed diabetes, and heart problems were identified based on the number of affirmative responses to symptoms indicative of these diseases. For each of these two diseases, only three questions were asked by the interviewer to decide if the respondent was labelled to have undiagnosed diabetes or an undiagnosed heart problem. However, these two diseases are commonly diagnosed based on a combination of clinical symptoms, physical examination, and further laboratory and imaging investigations, rather than from clinical symptoms alone. Hence, the ascertainment of multimorbidity in this study is of doubtful reliability.

Thirdly, the statistical tests used to identify the patterns of associative multimorbidity were varied [26]. Amongst the eight articles included, four different statistical methods were used—exploratory factor analysis, hierarchical cluster analysis, ratio observed/expected, and logistic regression.

Lastly, variations in the age of the study populations could have added an element of age bias which resulted in difficulty when comparing among the studies. Additionally, while some of the studies stratified their results by age, most of them did not, further elaborating the point of a lack of uniform methodology. Out of the eight studies, three articles stratified their analysis by age. Their study populations were ≥18 years old [20], ≥40 years old [18], and ≥60 years old [21]. The remaining five articles did not stratify their analysis by age. They included individuals ≥ 18 years old [17, 23], ≥50 years old [24], and ≥60 years old [19, 22]. As majority of the studies did not consider this variable, an element of age bias could have influenced the diseases identified amongst the study population and hence affected the patterns derived as well. While it is fair to assume that older patients are more susceptible to multimorbidity, it should not be limited to age alone [27]. Multimorbidity also affects a significant proportion of the younger population [28] and an unbalanced focus on the older population would result in multimorbidity patterns which are not truly representative of the general population.

### 4.2. Summary of Associative Multimorbidity Patterns

This segment focuses on disease clusters derived from six out of the eight articles selected [17, 19, 21–24]. The other two studies [18, 20] focused on dyadic/triadic disease combinations and not disease clusters and were therefore excluded. In general, disease clusters derived from each of the six articles which have commonalities were eventually categorized to five distinct patterns in this systematic review. The “cardiopulmonary-mental-degenerative disorders” described by Wang et al. [19] included many diseases and are not a discriminatory way of categorization. However, we classified “cardiopulmonary-mental-degenerative disorders” into one or more of the five proposed multimorbidity patterns as long as the diseases described by Wang et al. [19] fit the proposed patterns. While this may not be ideal, our overarching purpose was to present the multimorbidity patterns in this systematic review in an inclusive way without excluding potential links among diseases. We noted that none of the six articles which described associative multimorbidity patterns was completely concordant with each other and this observation was similarly mentioned in the systematic review by Prados-Torres et al. [11].

The five associative multimorbidity patterns identified in this systematic review included cardiovascular and metabolic diseases, mental health problems, degenerative diseases, pulmonary diseases, and cancer diseases. Out of these five patterns, two of them (cardiovascular and metabolic diseases and mental health problems) were similar to that found by Prados-Torres et al. [11]. The differences in the associative multimorbidity patterns obtained from Prados-Torres' study and this systematic review are likely multifactorial. Differences between the Western and Asian population due to cultural, genetic, or unknown reasons may be a contributing factor. Another key reason could be the heterogenicity of the methodologies.

Amongst the five patterns, two of them (cardiovascular and metabolic diseases and degenerative diseases) were reported by all the six selected articles. This showed that diseases such as those included in the cardiovascular and metabolic groups were some of the most prevalent diseases in most populations [29]. The clustering of degenerative diseases is explained by age-related changes. For example, aging is commonly associated with eye problems such as age-related macular degeneration, cataract and presbyopia, hearing problems such as presbycusis, arthritis due to age-related degeneration of joints, and memory-related conditions. In order to have a better understanding of associative multimorbidity patterns found in this systematic review, more studies using an agreed-upon methodology and definition of multimorbidity should be conducted in Asia.

### 4.3. Study Limitations

Language restrictions were one of the limitations as studies which were written in other Asian languages such as Chinese, Japanese, and Korean could have been missed out. Due to the small number of studies included in this systematic review, the patterns identified may not be truly representative of associative multimorbidity patterns in Asia as a whole.

## 5. Conclusion and Future Research

This systematic review identified five common patterns of associative multimorbidity in Asia. Similar multimorbidity patterns like “cardiovascular and metabolic diseases” and “mental health problems” were found in the Western world. There was much heterogeneity in the definition of multimorbidity and statistical methodology in the selected articles. Researchers embarking on future studies looking at patterns of associative multimorbidity need to work collaboratively to achieve a consensus on the definition of multimorbidity and align statistical methodologies. This will allow reliable and distinct multimorbidity patterns to be identified in Asia so that ultimately, clinical practice guidelines on multimorbidity can be developed to contextualize management of the unique patterns of associative multimorbidity seen in Asia.

## Figures and Tables

**Table 1:** Characteristics of the selected articles.

	Author, year, country	Population	Study design; sampling strategies	Data source: data collection	Definition of multimorbidity	Number of chronic diseases	List of chronic diseases
1	Garin et al., 2015, China and India [17]	General population; 14794 individuals ≥ 18 years old	Cross-sectional; multistage clustered sampling	Study on Global AGEing and Adult Health (SAGE); structured interviews and physician's assessment	≥2 chronic diseases	12	Angina, arthritis, asthma, cataract, chronic obstructive pulmonary disease (COPD), depression, diabetes, edentulism, hypertension, cognitive impairment, obesity, and stroke
		General population; 11230 individuals ≥ 18 years old	Cross-sectional; multistage clustered sampling	Study on Global AGEing and Adult Health (SAGE); structured interviews and physician's assessment	≥2 chronic diseases	12	Angina, arthritis, asthma, cataract, chronic obstructive pulmonary disease (COPD), depression, diabetes, edentulism, hypertension, cognitive impairment, obesity, and stroke

2	Hussain et al., 2015, Indonesia [18]	General population; 9438 individuals ≥ 40 years old	Cross-sectional; multistage clustered sampling	Indonesian Family Life Survey (IFLS-4); self-reported questionnaire and trained nurse's assessment	≥2 chronic diseases	15	Hypertension, hypercholesterolaemia, cardiac diseases, obesity (body mass index ≥ 30), arthritis, vision abnormality, uric acid/gout, depression, chronic respiratory diseases, diabetes mellitus, hearing problem, stroke, liver disease, tuberculosis, and cancer

3	Wang et al., 2015, China [19]	General population; 1480 individuals ≥ 60 years old	Cross-sectional; cluster sampling	Confucius Hometown Aging Project (CHAP); structured interviews, clinical examinations, and laboratory tests	≥2 chronic diseases	16	Hypertension, diabetes, obesity, dyslipidemia, thyroid dysfunction, coronary heart disease, arrhythmia, eye problems, chronic obstructive pulmonary disease or asthma, depression, cognitive impairment, heart failure, stroke, hearing disorders, tumor, and arthritis

4	Wang et al., 2015, China [20]	General population; 21435 individuals 18–79 years old	Cross-sectional; multistage stratified cluster sampling	Jilin Provincial Chronic Disease Survey; structured interviews and clinical examination on BMI	≥2 chronic diseases in the past 12 months	18	Anemia, diabetes mellitus, severe vision reduction, hypertension, ischemic heart disease, cerebrovascular disease, chronic nasopharyngitis, chronic lower respiratory disease, chronic gastroenteritis/peptic ulcer, liver disease, cholecystitis/gallstones, arthritis, chronic low back pain, chronic nephritis, urolithiasis, prostatic hyperplasia (male), disorders of breast (female), and pelvic inflammatory disease (female)

5	Gu et al., 2017, China [21]	Primary care; 2452 individuals ≥ 60 years old	Cross-sectional; stratified cluster sampling	Community Health Service, Nanjing, Jiangsu province; structured interviews, self-reported questionnaire, and physician's assessment	≥2 chronic diseases	13	Hypertension, diabetes, joint disease, cataract, hearing disorder, dyslipidemia, coronary heart disease, stroke, kidney disease, gastrointestinal disease, lung disease, liver disease, and cancer

6	Gu et al., 2018, China [22]	Primary care; 411 individuals ≥ 60 years old	Cross-sectional; simple random sampling from Gu et al., 2016	Community Health Service, Nanjing, Jiangsu province; structured interviews, self-reported questionnaire, and physician's assessment	≥2 chronic diseases	13	Hypertension, diabetes, joint disease, cataract, hearing disorder, dyslipidemia, coronary heart disease, stroke, kidney disease, gastrointestinal disease, lung disease, liver disease, and cancer

7	Aoki et al., 2018, Japan [23]	General population; 3256 individuals 18-84 years old	Cross-sectional; quota sampling from residents' panel	Norm study; self-reported questionnaire	≥2 chronic diseases	17	Hypertension, diabetes, dyslipidemia, stroke, cardiac diseases, chronic respiratory diseases, digestive diseases, kidney diseases, urologic diseases, arthritis or rheumatism, lumbar diseases, neurologic diseases, mental disorders, endocrine diseases, malignancy, vision abnormalities, and skin diseases

8	Yao et al., 2019, China [24]	General population; 19841 individuals ≥ 50 years old	Cross-sectional; multistage sampling	China Health and Retirement Longitudinal Study (CHARLS); structured interviews and self-reported questionnaire	≥2 chronic diseases	15	Hypertension, dyslipidemia, diabetes or high blood sugar, cancer or malignant tumor, chronic lung disease, liver disease, heart problems, stroke, kidney disease, stomach or other digestive disease, emotional, nervous or psychiatric problems, memory-related disease, arthritis or rheumatism, hip fracture, and vision impairment

**Table 2:** Statistical methodological approaches for the determination of associative multimorbidity.

	Author, year, country	Statistical analysis	Patterns of multimorbidity	Stratification variables	Proximity measures	Type of clustering algorithm	Pattern number determination criteria (statistical)
1	Garin et al., 2015, China and India [17]	Exploratory factor analysis	Disease clusters	No	Tetrachoric correlation matrix	NS	Scree plot test and parallel analysis
		Exploratory factor analysis	Disease clusters	No	Tetrachoric correlation matrix	NS	Scree plot test and parallel analysis

2	Hussain et al., 2015, Indonesia [18]	Observed/expected ratio	Dyads and triads	Yes (by age and sex)	NA	Conditional probability	Chi-square test

3	Wang et al., 2015, China [19]	Observed/expected ratio	Dyads	No	NA	NS	Logistic regression
		Exploratory factor analysis	Disease clusters	No	Tetrachoric correlation matrix	NS	Eigenvalues ≥ 1.0

4	Wang et al., 2015, China [20]	Logistic regression	Dyads	Yes (by age and sex)	NA	Conditional probability	Adjusted odds ratio > 3.0

5	Gu et al., 2017, China [21]	Observed/expected ratio	Dyads	Yes (by age and sex)	NA	Conditional probability	Logistic regression
		Exploratory factor analysis	Disease clusters	Yes (by age and sex)	Correlation matrix	Principal factor method	Eigenvalues ≥ 1.0

6	Gu et al., 2018, China [22]	Exploratory factor analysis	Disease clusters	No	Tetrachoric correlation matrix	Principal factor method	Eigenvalues ≥ 1.0

7	Aoki et al., 2018, Japan [23]	Exploratory factor analysis	Disease clusters	No	Tetrachoric/polychoric correlation matrix	NS	Scree plot test and parallel analysis

8	Yao et al., 2019, China [24]	Hierarchical cluster analysis	Disease clusters	Yes (by sex and residential regions)	Yule's Q distance	Average linkage	NS

NS: not stated.

**Table 3:** Risk of bias assessment for selected articles using the Modified Newcastle-Ottawa Scale.

	Author, year, country	Representativeness of sample	Ascertainment of multimorbidity	Appropriateness of statistical method
1	Garin et al., 2015, China and India [17]	Low risk	Low risk	Low risk
		Low risk	Low risk	Low risk

2	Hussain et al., 2015, Indonesia [18]	Low risk	Medium risk	Low risk

3	Wang et al., 2015, China [19]	Low risk	Low risk	Low risk

4	Wang et al., 2015, China [20]	Low risk	Low risk	Low risk

5	Gu et al., 2017, China [21]	Low risk	Low risk	Low risk

6	Gu et al., 2018, China [22]	Low risk	Low risk	Low risk

7	Aoki et al., 2018, Japan [23]	Low risk	High risk	Low risk

8	Yao et al., 2019, China [24]	Low risk	High risk	Low risk

**Table 4:** Patterns of multimorbidity identified from selected articles.

	Multimorbidity patterns	Author, year, country	Patterns identified	Diseases included	Diseases excluded
1	*Cardiovascular and metabolic diseases*	Garin et al., 2015, China and India [17]	Cardiorespiratory	Angina	Asthma, COPD
			Metabolic	Diabetes, obesity, and hypertension	
		Wang et al., 2015, China [19]	Cardiopulmonary-mental-degenerative disorders	Heart failure, arrhythmia, and coronary heart diseases	COPD, asthma, depression, eye problems, and hearing disorders
			Cerebrovascular-metabolic disorders	Stroke, hypertension, diabetes, dyslipidemia, and obesity	
		Gu et al., 2017, China [21]	Cardiovascular and metabolic disorders	Hypertension, diabetes, coronary heart disease, kidney diseases, and dyslipidemia	
		Gu et al., 2018, China [22]	Cardiovascular and metabolic disorders	Hypertension, diabetes, and coronary heart disease, kidney diseases, and dyslipidemia	
		Aoki et al., 2018, Japan [23]	Cardiovascular/renal/metabolic	Hypertension, diabetes, coronary heart disease, stroke, kidney diseases, and dyslipidemia	
		Yao et al., 2019, China [24]	Vascular-metabolic	Hypertension, dyslipidemia, diabetes, and stroke	
			Hepatorenal	Kidney disease	Liver disease

2	*Mental health problems*	Garin et al., 2015, China and India [17]	Mental-articular	Depression	Arthritis
		Wang et al., 2015, China [19]	Cardiopulmonary-mental-degenerative disorders	Depression	Heart failure, arrhythmia, coronary heart diseases, COPD, asthma, eye problems, and hearing disorders
		Aoki et al., 2018, Japan [23]	Neuropsychiatric	Mental disorders	Neurologic diseases
		Yao et al., 2019, China [24]	Cognitive-emotional	Emotional, nervous, or psychiatric problems	Memory-related disease

3	*Degenerative diseases*	Garin et al., 2015, China and India [17]	Mental-articular	Arthritis	Depression
		Wang et al., 2015, China [19]	Cardiopulmonary-mental-degenerative disorders	Eye problems, hearing disorders	Heart failure, arrhythmia, coronary heart diseases, COPD, and asthma, depression
		Gu et al., 2017, China [21]	Degenerative disorders	Cataract, joint disease, and hearing disorder	Cancer
		Gu et al., 2018, China [22]	Degenerative disorders	Cataract, joint disease, and hearing disorder	Cancer
		Aoki et al., 2018, Japan [23]	Skeletal/articular/digestive	Arthritis or rheumatism, lumbar diseases	Digestive diseases
		Yao et al., 2019, China [24]	Cognitive-emotional	Memory-related disease	Emotional, nervous, or psychiatric problems

4	*Pulmonary diseases*	Garin et al., 2015, China and India [17]	Cardiorespiratory	Asthma, COPD	Angina
		Wang et al., 2015, China [19]	Cardiopulmonary-mental-degenerative disorders	COPD, asthma	Heart failure, arrhythmia, coronary heart diseases, depression, eye problems, and hearing disorders
		Gu et al., 2017, China [21]	Digestive and respiratory disorders	Lung diseases	Gastrointestinal diseases, liver diseases
		Gu et al., 2018, China [22]	Digestive and respiratory disorders	Lung diseases	Gastrointestinal diseases, liver diseases
		Aoki et al., 2018, Japan [23]	Respiratory/dermal	Chronic respiratory diseases	Skin diseases

5	*Cancer diseases*	Gu et al., 2017, China [21]	Degenerative disorders	Cancer	Cataract, joint disease, and hearing disorder
		Gu et al., 2018, China [22]	Degenerative disorders	Cancer	Cataract, joint disease, and hearing disorder
		Aoki et al., 2018, Japan [23]	Malignant/digestive/urologic	Malignancy	Digestive diseases, urologic diseases

## Data Availability

No data were used to support this study.

## Supplementary Materials

Supplementary 1 Appendix S1: search strategy from Medical Literature Analysis and Retrieval System Online (MEDLINE (Ovid)), Excerpta Medica Database (EMBASE), Cumulative Index to Nursing and Allied Health Literature (CINAHL), Web of Science (Clarivate Analytics), and Scopus (Elsevier) on April 22, 2019.

Supplementary 2 Appendix S2: risk of the bias tool using the Modified Newcastle-Ottawa Scale.

## References

[1] Prados-Torres A., Poblador-Plou B., Calderón-Larrañaga A. (2012). Multimorbidity patterns in primary care: interactions among chronic diseases using factor analysis. *PLoS One*.

[2] Fortin M., Bravo G., Hudon C., Vanasse A., Lapointe L. (2005). Prevalence of multimorbidity among adults seen in family practice. *Annals of Family Medicine*.

[3] Spencer-Bonilla G., Quiñones A. R., Montori V. M. (2017). Assessing the burden of treatment. *Journal of General Internal Medicine*.

[4] Ong K., Lee P. S. S., Lee E. (2019). Patient-centred and not disease-focused: a review of guidelines and multimorbidity. *Singapore Medical Journal*.

[5] Cassell A., Edwards D., Harshfield A. (2018). The epidemiology of multimorbidity in primary care: a retrospective cohort study. *The British Journal of General Practice*.

[6] Salisbury C., Johnson L., Purdy S., Valderas J. M., Montgomery A. A. (2011). Epidemiology and impact of multimorbidity in primary care: a retrospective cohort study. *The British Journal of General Practice*.

[7] Tinetti M. E., Bogardus S. T., Agostini J. V. (2004). Potential pitfalls of disease-specific guidelines for patients with multiple conditions. *New England Journal of Medicine*.

[8] Multimorbidity: clinical assessment and management (2016). *NICE guideline*.

[9] Olson J. E., Takahashi P. Y., St. Sauver J. M. (2018). Understanding the patterns of multimorbidity. *Mayo Clinic Proceedings*.

[10] Verbrugge L. M., Lepkowski J. M., Imanaka Y. (1989). Comorbidity and its impact on disability. *The Milbank Quarterly*.

[11] Prados-Torres A., Calderón-Larrañaga A., Hancco-Saavedra J., Poblador-Plou B., van den Akker M. (2014). Multimorbidity patterns: a systematic review. *Journal of Clinical Epidemiology*.

[12] Liberati A., Altman D. G., Tetzlaff J. (2009). The PRISMA statement for reporting systematic reviews and meta-analyses of studies that evaluate healthcare interventions: explanation and elaboration. *BMJ*.

[13] Tugwell P., Knottnerus J. A. (2019). Multimorbidity and comorbidity are now separate MESH headings. *Journal of Clinical Epidemiology*.

[14] Nicholson K., Makovski T. T., Griffith L. E., Raina P., Stranges S., van den Akker M. (2019). Multimorbidity and comorbidity revisited: refining the concepts for international health research. *Journal of Clinical Epidemiology*.

[15] Fortin M., Stewart M., Poitras M.-E., Almirall J., Maddocks H. (2012). A systematic review of prevalence studies on multimorbidity: toward a more uniform methodology. *Annals of Family Medicine*.

[16] Alshabanat A., Zafari Z., Albanyan O., Dairi M., Fitz Gerald J. M. (2015). Asthma and COPD overlap syndrome (ACOS): a systematic review and meta analysis. *PLoS One*.

[17] Garin N., Koyanagi A., Chatterji S. (2015). Global multimorbidity patterns: a cross-sectional, population-based, multi-country study. *The Journals of Gerontology: Series A*.

[18] Hussain M. A., Huxley R. R., Al M. A. (2015). Multimorbidity prevalence and pattern in Indonesian adults: an exploratory study using national survey data. *BMJ Open*.

[19] Wang R., Yan Z., Liang Y. (2015). Prevalence and patterns of chronic disease pairs and multimorbidity among older Chinese adults living in a rural area. *PLoS One*.

[20] Wang S. B., D'Arcy C., Yu Y. Q. (2015). Prevalence and patterns of multimorbidity in northeastern China: a cross-sectional study. *Public Health*.

[21] Gu J., Chao J., Chen W. (2017). Multimorbidity in the community-dwelling elderly in urban China. *Archives of Gerontology and Geriatrics*.

[22] Gu J., Chao J., Chen W. (2018). Multimorbidity and health-related quality of life among the community-dwelling elderly: a longitudinal study. *Archives of Gerontology and Geriatrics*.

[23] Aoki T., Yamamoto Y., Ikenoue T., Onishi Y., Fukuhara S. (2018). Multimorbidity patterns in relation to polypharmacy and dosage frequency: a nationwide, cross-sectional study in a Japanese population. *Scientific Reports*.

[24] Yao S. S., Cao G. Y., Han L. (2019). Prevalence and Patterns of Multimorbidity in a Nationally Representative Sample of Older Chinese: Results from the China Health and Retirement Longitudinal Study. *The Journals of Gerontology: Series A*.

[25] Althubaiti A. (2016). Information bias in health research: definition, pitfalls, and adjustment methods. *Journal of Multidisciplinary Healthcare*.

[26] Ng S. K., Tawiah R., Sawyer M., Scuffham P. (2018). Patterns of multimorbid health conditions: a systematic review of analytical methods and comparison analysis. *International Journal of Epidemiology*.

[27] Nobili A., Garattini S., Mannucci P. (2011). Multiple diseases and polypharmacy in the elderly: challenges for the internist of the third millennium. *Journal of comorbidity*.

[28] Menditto E., Gimeno Miguel A., Moreno Juste A. (2019). Patterns of multimorbidity and polypharmacy in young and adult population: systematic associations among chronic diseases and drugs using factor analysis. *PLoS One*.

[29] Organization WH *World Health Statistics 2018: monitoring health for the SDGs*.

