# Supplementary material for: A Systematic Review of the Patterns of Associative Multimorbidity in Asia
Source: Biomed Res Int. 2021 Jul 3;2021:6621785. doi: 10.1155/2021/6621785 (PMC8277911; doi:10.1155/2021/6621785)
Supplement: Supplementary 1 — Appendix S1: search strategy from Medical Literature Analysis and Retrieval System Online (MEDLINE (Ovid)), Excerpta Medica Database (EMBASE), Cumulative Index to Nursing and Allied Health Literature (CINAHL), Web of Science (Clarivate Analytics), and Scopus (Elsevier) on April 22, 2019. [file 6621785.f1.docx]

**APPENDIX S1**

**Search strategy**

Database: Medline(Ovid)

First year searched: since inception

Last year searched: 2019

Search conducted in April 22, 2019

Who conducted the search: Authors ZJW and SSR

1. exp Comorbidity/ or Multiple Chronic Conditions/

2. (Comorbid* or co-morbid* or Multimorbid* or multi-morbid* or Multidisease* or multi-disease* or ((cooccur* or co-occur* or coexist* or co-exist* or multipl*) adj2 (disease* or ill* or care or condition* or disorder* or medication* or symptom* or syndrom*))).ab,ti,kw.

3. (chronic adj2 (disease* or ill* or care or condition* or disorder* or health* or medication* or syndrome* or symptom*)).ab,ti,kw.

4. 1 or 2 or 3

5. (Asia* or Kazakhstan or Uzbekistan or China or Chinese or Hong Kong or Japan* or Korea* or Taiwan* or Mongolia* or Bangladesh* or India* or Iran* or Pakistan* or Bhutan or Nepal* or Sri Lanka or Maldives or Malaysia* or Singapor* or Thai* or Borneo or Brunei or Cambodia* or Indochina or Indonesia* or Laos or Mekong Valley or Myanmar or Philippin* or Timor-Leste or Vietnam* or Afghanistan or Iraq or Bahrain or Yemen or Palestine* or Egypt or Israel or Jordan or Kuwait or Lebanon or Oman or Qatar or Saudi Arabia* or Syrian* or Turkey or United Arab Emirates).ab,ti,kw.

6. exp asia/

7. 5 or 6

8. exp Primary Health Care/ or exp Physicians, Primary Care/ or exp General Practitioners/ or exp Physicians, Family/ or "continuity of patient care"/ or community health services/

9. (((primary or family or general or generalist* or communit*) adj2 (care* or medical* or health* or clinic* or practitioner* or doctor* or practitioner* or practice)) or population).ab,ti,kw.

10. 8 or 9

11. exp prevalence/ or exp association/ or exp cluster analysis/ or Factor Analysis, Statistical/

12. (prevalence or pattern* or cluster* or associati* or "observed/expected" or "O/E" or "factor analysis").ab,ti,kw.

13. 11 or 12

14. 4 and 7 and 10 and 13Database: Embase

First year searched: since inception

Last year searched: 2019

Search conducted in April 22, 2019

Who conducted the search: Authors ZJW and SSR

1. 'comorbidity'/exp OR 'comorbidity' OR 'multiple chronic conditions'/exp OR 'multiple chronic conditions'

2. comorbid*:ab,ti,kw OR 'co morbid*':ab,ti,kw OR multimorbid*:ab,ti,kw OR 'multi morbid*':ab,ti,kw OR multidisease*:ab,ti,kw OR 'multi disease*':ab,ti,kw OR (((cooccur* OR 'co occur*' OR coexist* OR 'co exist*' OR multipl*) NEAR/2 (disease* OR ill* OR care OR condition* OR disorder* OR medication* OR symptom* OR syndrom*)):ab,ti,kw)

3. (chronic NEAR/2 (disease* OR ill* OR care OR condition* OR disorder* OR health* OR medication* OR syndrome* OR symptom*)):ab,ti,kw

4. #1 OR #2 OR #3

5. 'asia'/exp

6. asia*:ab,ti,kw OR kazakhstan:ab,ti,kw OR uzbekistan:ab,ti,kw OR china:ab,ti,kw OR chinese:ab,ti,kw OR 'hong kong':ab,ti,kw OR japan*:ab,ti,kw OR korea*:ab,ti,kw OR taiwan*:ab,ti,kw OR mongolia*:ab,ti,kw OR bangladesh*:ab,ti,kw OR india*:ab,ti,kw OR iran*:ab,ti,kw OR pakistan*:ab,ti,kw OR bhutan:ab,ti,kw OR nepal*:ab,ti,kw OR 'sri lanka':ab,ti,kw OR maldives:ab,ti,kw OR malaysia*:ab,ti,kw OR singapor*:ab,ti,kw OR thai*:ab,ti,kw OR borneo:ab,ti,kw OR brunei:ab,ti,kw OR cambodia*:ab,ti,kw OR indochina:ab,ti,kw OR indonesia*:ab,ti,kw OR laos:ab,ti,kw OR 'mekong valley':ab,ti,kw OR myanmar:ab,ti,kw OR philippin*:ab,ti,kw OR 'timor-leste':ab,ti,kw OR vietnam*:ab,ti,kw OR afghanistan:ab,ti,kw OR iraq:ab,ti,kw OR bahrain:ab,ti,kw OR yemen:ab,ti,kw OR palestine*:ab,ti,kw OR egypt:ab,ti,kw OR israel:ab,ti,kw OR jordan:ab,ti,kw OR kuwait:ab,ti,kw OR lebanon:ab,ti,kw OR oman:ab,ti,kw OR qatar:ab,ti,kw OR 'saudi arabia*':ab,ti,kw OR syrian*:ab,ti,kw OR turkey:ab,ti,kw OR 'united arab emirates':ab,ti,kw

7. #5 OR #6

8. 'primary health care'/exp OR 'physicians, primary care'/exp OR 'general practitioners'/exp OR 'physicians, family'/exp

9. (((primary OR family OR general OR generalist* OR communit*) NEAR/2 (care* OR medical* OR health* OR clinic* OR practitioner* OR doctor* OR practitioner* OR practice)):ab,ti,kw) OR population:ab,ti,kw

10. #8 OR #9

11. 'prevalence'/exp OR 'association'/exp OR 'cluster analysis'/exp OR 'factor analysis'/exp

12. prevalence:ab,ti,kw OR pattern*:ab,ti,kw OR cluster*:ab,ti,kw OR associati*:ab,ti,kw OR 'observed/expected':ab,ti,kw OR 'o/e':ab,ti,kw OR factor*:ab,ti,kw

13. #11 OR #12

14. #4 AND #7 AND #10 AND #13

Database: Cumulative Index to Nursing and Allied Health Literature (CINAHL)

First year searched: since inception

Last year searched: 2019

Search conducted in April 22, 2019

Who conducted the search: Authors ZJW and SSR

1. (MH "Comorbidity")

2. AB ( (Comorbid* or co-morbid* or Multimorbid* or multi-morbid* or Multidisease* or multi-disease* or ((cooccur* or co-occur* or coexist* or co-exist* or multipl*) N2 (disease* or ill* or care or condition* or disorder* or medication* or symptom* or syndrom*))) ) OR TI ( (Comorbid* or co-morbid* or Multimorbid* or multi-morbid* or Multidisease* or multi-disease* or ((cooccur* or co-occur* or coexist* or co-exist* or multipl*) N2 (disease* or ill* or care or condition* or disorder* or medication* or symptom* or syndrom*))) )

3. TI ( (chronic N2 (disease* or ill* or care or condition* or disorder* or health* or medication* or syndrome* or symptom*)) ) OR AB ( (chronic N2 (disease* or ill* or care or condition* or disorder* or health* or medication* or syndrome* or symptom*)) )

4. S1 OR S2 OR S3

5. (MH "Asia+")

6. TI ( (Asia* or Kazakhstan or Uzbekistan or China or Chinese OR Hong Kong or Japan* or Korea* or Taiwan* OR Mongolia* or Bangladesh* or India* OR Iran* OR Pakistan* OR Bhutan OR Nepal* OR Sri Lanka or Maldives OR Malaysia* or Singapor* or Thai* or Borneo or Brunei or Cambodia* OR Indochina OR Indonesia* OR Laos OR Mekong Valley OR Myanmar or Philippin* OR Timor-Leste OR Vietnam* OR Afghanistan OR Iraq OR Bahrain OR Yemen OR Palestine* OR Egypt OR Israel OR Jordan OR Kuwait OR Lebanon OR Oman OR Qatar OR Saudi Arabia* OR Syrian* OR Turkey OR United Arab Emirates) ) OR AB ( (Asia* or Kazakhstan or Uzbekistan or China or Chinese OR Hong Kong or Japan* or Korea* or Taiwan* OR Mongolia* or Bangladesh* or India* OR Iran* OR Pakistan* OR Bhutan OR Nepal* OR Sri Lanka or Maldives OR Malaysia* or Singapor* or Thai* or Borneo or Brunei or Cambodia* OR Indochina OR Indonesia* OR Laos OR Mekong Valley OR Myanmar or Philippin* OR Timor-Leste OR Vietnam* OR Afghanistan OR Iraq OR Bahrain OR Yemen OR Palestine* OR Egypt OR Israel OR Jordan OR Kuwait OR Lebanon OR Oman OR Qatar OR Saudi Arabia* OR Syrian* OR Turkey OR United Arab Emirates) )

7. S5 OR S6

8. (MH "Primary Health Care") OR (MH "Physicians, Family") OR (MH "Family Practice")

9. TI (population) OR AB (population)

10. S8 OR S9

11. AB ( ((primary or family OR general or generalist* or communit*) N2 (care* or medical* or health* or clinic* or practitioner* or doctor* or practitioner* or practice)) ) OR TI ( ((primary or family OR general or generalist* or communit*) N2 (care* or medical* or health* or clinic* or practitioner* or doctor* or practitioner* or practice)) )

12. S10 OR S11

13. (MH "Factor Analysis") OR (MH "Cluster Analysis")

14. (MH "Prevalence")

15. S13 OR S14

16. TI ( prevalence OR pattern* OR cluster* OR associati* OR "observed/expected" or "O/E" or factor* ) OR AB ( prevalence OR pattern* OR cluster* OR associati* OR "observed/expected" or "O/E" or factor* )

17. S15 OR S16

18. S4 AND S7 AND S12 AND S17

Database: Web of Science

First year searched: since inception

Last year searched: 2019

Search conducted in April 22, 2019

Who conducted the search: Authors ZJW and SSR

1. TS = ((Comorbid* or co-morbid* or Multimorbid* or multi-morbid* or Multidisease* or multi-disease* or ((cooccur* or co-occur* or coexist* or co-exist* or multipl*) NEAR/2 (disease* or ill* or care or condition* or disorder* or medication* or symptom* or syndrom*))))

2. TS = (chronic near/2 (disease* or ill* or care or condition* or disorder* or health* or medication* or syndrome* or symptom*))

3. #2 OR #1

4. TS = (Asia* or Kazakhstan or Uzbekistan or China or Chinese OR Hong Kong or Japan* or Korea* or Taiwan* OR Mongolia* or Bangladesh* or India* OR Iran* OR Pakistan* OR Bhutan OR Nepal* OR Sri Lanka or Maldives OR Malaysia* or Singapor* or Thai* or Borneo or Brunei or Cambodia* OR Indochina OR Indonesia* OR Laos OR Mekong Valley OR Myanmar or Philippin* OR Timor-Leste OR Vietnam* OR Afghanistan OR Iraq OR Bahrain OR Yemen OR Palestine* OR Egypt OR Israel OR Jordan OR Kuwait OR Lebanon OR Oman OR Qatar OR Saudi Arabia* OR Syrian* OR Turkey OR United Arab Emirates)

5. TS = (((primary or family OR general or generalist* or communit*) near/2 (care* or medical* or health* or clinic* or practitioner* or doctor* or practitioner* or practice)) or population)

6. TS = (prevalence OR pattern* OR cluster* OR associati* OR "observed/expected" or "O/E" or “factor analysis”)

7. #6 AND #5 AND #4 AND #3

Database: Scopus

First year searched: since inception

Last year searched: 2019

Search conducted in April 22, 2019

Who conducted the search: Authors ZJW and SSR

( ( TITLE-ABS-KEY ( "multiple chronic condition*" ) )  OR ( TITLE-ABS-KEY ( comorbid* OR co-morbid* OR multimorbid*  OR multi-morbid* OR multidisease* OR multi-disease* OR ( ( cooccur*  OR co-occur* OR coexist* OR co-exist* OR multipl* ) W/2 ( disease* OR  ill* OR care OR condition* OR disorder* OR medication* OR symptom* OR syndrom* ) ) ) )  OR ( TITLE-ABS-KEY ( chronic W/2 ( disease* OR ill* OR care OR condition* OR disorder* OR  health* OR medication* OR syndrome* OR symptom* ) ) ) ) AND ( TITLE-ABS-KEY ( ( primary OR family  OR general OR generalist* OR communit* ) W/2 ( care* OR medical* OR health* OR clinic* OR practitioner*  OR doctor* OR practitioner* OR practice ) OR population) ) AND ( TITLE-ABS-KEY ( prevalence OR pattern* OR cluster*  OR associati* OR "observed/expected" OR "O/E" OR "factor analysis" ) ) AND ( TITLE-ABS-KEY ( asia* OR kazakhstan OR uzbekistan  OR china OR chinese OR "Hong Kong" OR japan* OR korea* OR taiwan* OR mongolia* OR bangladesh* OR india* OR iran* OR pakistan*  OR bhutan OR nepal* OR "Sri Lanka" OR maldives OR malaysia* OR singapor* OR thai* OR borneo OR brunei OR cambodia* OR indochina  OR indonesia* OR laos OR "Mekong Valley" OR myanmar OR philippin* OR timor-leste OR vietnam* OR afghanistan OR iraq OR bahrain OR yemen  OR palestine* OR egypt OR israel OR jordan OR kuwait OR lebanon OR oman OR qatar OR "Saudi Arabia*" OR syrian* OR turkey OR "United Arab Emirates" ) )
