# Supplementary material for: A Systematic Review of the Patterns of Associative Multimorbidity in Asia
Source: Biomed Res Int. 2021 Jul 3;2021:6621785. doi: 10.1155/2021/6621785 (PMC8277911; doi:10.1155/2021/6621785)
Supplement: Supplementary 2 — Appendix S2: risk of the bias tool using the Modified Newcastle-Ottawa Scale. [file 6621785.f2.docx]

APPENDIX S2

| **Category** | **Description** |
| --- | --- |
| **Representativeness of Sample** | This item assesses the representativeness of the sample in the community, not from some general population.   - 1. Truly representative* (e.g. everyone from the database)   2. Somewhat representative* (with at least 2 criteria but selection method was convincing due to random sampling)   3. Selected group (e.g. only certain socio-economic groups or areas)   4. No description of sampling strategy |
| **Ascertainment of Multimorbidity** | This item assesses the method by which multimorbidity was confirmed.   1. Secure record* (e.g. interview, physical exam, laboratory test by physician) 2. Structured interview* (e.g. interviewer-administered questionnaire) 3. Written self-report (e.g. mailed survey, if items are unable to be confirmed by objective measure) 4. No description / Other |
| **Appropriateness of Statistical Test** | This item assesses the appropriateness of statistical method used to prove non-random association between diseases. All statistical methods were deemed appropriate, including the statistical methods stated by Garin^^[[1]](#footnote-1)^^.   1. Exploratory Factor Analysis* 2. Cluster Analysis* 3. Multiple Correspondence Analysis* 4. Ratio Observed/Expected* 5. Other statistical methods not stated by Garin (1)* 6. No/poor description |

* Low risk of bias

1. Global Multimorbidity Patterns: A Cross-Sectional, Population-Based, Multi-Country Study - Appendix 2 [↑](#footnote-ref-1)
